# Cross-Validation of Functional MRI and Paranoid-Depressive Scale: Results From Multivariate Analysis

**DOI:** 10.3389/fpsyt.2019.00869

**Published:** 2019-11-25

**Authors:** Drozdstoy Stoyanov, Sevdalina Kandilarova, Rositsa Paunova, Javier Barranco Garcia, Adeliya Latypova, Ferath Kherif

**Affiliations:** ^1^Department of Psychiatry and Medical Psychology, Faculty of Medicine, Medical University of Plovdiv, Plovdiv, Bulgaria; ^2^Laboratory for Research in Neuroimaging, Department of Clinical Neuroscience, Lausanne University Hospital and University of Lausanne, Lausanne, Switzerland

**Keywords:** validation, psychopathology, machine learning, functional MRI, classification

## Abstract

**Introduction:** There exists over the past decades a constant debate driven by controversies in the validity of psychiatric diagnosis. This debate is grounded in queries about both the validity and evidence strength of clinical measures.

**Materials and Methods:** The objective of the study is to construct a bottom-up unsupervised machine learning approach, where the brain signatures identified by three principal components based on activations yielded from the three kinds of diagnostically relevant stimuli are used in order to produce cross-validation markers which may effectively predict the variance on the level of clinical populations and eventually delineate diagnostic and classification groups. The stimuli represent items from a paranoid-depressive self-evaluation scale, administered simultaneously with functional magnetic resonance imaging (fMRI).

**Results:** We have been able to separate the two investigated clinical entities – schizophrenia and recurrent depression by use of multivariate linear model and principal component analysis. Following the individual and group MLM, we identified the three brain patterns that summarized all the individual variabilities of the individual brain patterns.

**Discussion:** This is a confirmation of the possibility to achieve bottom-up classification of mental disorders, by use of the brain signatures relevant to clinical evaluation tests.

## Introduction

There exists over the past decades a constant debate driven by controversies in the validity of psychiatric diagnosis (1). This debate is grounded in queries about both the validity and evidence strength of clinical measures and the relevant classification and nomenclature systems (2) and eventually lead into crisis of confidence in psychiatry as medical discipline.

Those queries refer to a large extent to missing cross-validation of the clinical evaluation tools with data and explanatory models from neuroscience (3) and might be summarized in the following caveats.

- Normative and validation standards in psychopathology are fragmented from basic neuroscience, which applies different validation standards and procedures, both on statistical and conceptual levels.
- Fundamentally psychiatric clinical measures are constituted from narratives of the patient (self-assessment scales), the informant, and the expert (clinical rating scales), which are essentially comprised of subjective introspective and inter-subjective Likert scale items (4).
- Diagnostic entities in clinical psychiatry are not defined by biological signatures of disease as in the other medical disciplines, but with combinations and/or comparisons of those evaluation scales.

In our previous studies we have attempted to demonstrate the convergent and discriminative construct validity of the Depression Scale (5) and the functional magnetic resonance imaging (fMRI) signal by simultaneous administration of the items from the clinical scale as stimuli (6, 7). In those studies, we have employed neutral items from interest scale as contrast stimuli under block paradigm design. The t-contrasts on the second level of between-group comparison between patients with depression and healthy controls demonstrated significant differences in the activation of various brain regions during diagnostically significant scale items processing, contrasted with the processing of diagnostically neutral ones, notably in the left middle frontal gyrus, among others.

This paradigm has been further expanded by inclusion of paranoid items from Paranoid Depressive Scale (PD-S) by Von Zerssen and schizophrenia patients in order to investigate the contrast across different nosological groups and respective clinical measures (8). This model has been defined in top-down manner, from the clinical definition (psychiatric interview) to the corresponding brain activation determined by fMRI, administered simultaneously with clinical assessment scale (PD-S). Although certain encouraging results appeared on within-group level, they did not cross the statistical significance threshold on the between-group analysis level. We assumed that several factors undermine the translation of the functional MRI results to clinical measures in our data set. On one hand these disease entities might be assumed as a continuum of manifestation of one and the same underlying neurodegenerative or neuro-progressive process, as it is supported with reported abnormalities in the grey matter volume in patients with depression detected with voxel based morphometry (9–12). On the other hand, the included diagnoses may well represent discrete entities and the small number of recruited patients might be considered as confound in this study. Other caveats concern the innovative and non-conventional approach to the experimental paradigm design, which presents an issue for comparison with other studies in the field and the gender structure of the sample (8).

One critical premise of that model for translational validation is an exemplar instrumentalist validation (3), however including more robust biological reference measures. This approach is based on the assumption that scientific knowledge is instrumental: basically, it can provide us with suitable information about some limited domain of phenomena, and it explains and solves problems associated with that domain. In our case it would be instrumental to discriminate two clinical measurement constructs (paranoia and depression) with an incremental external validity operation, such as fMRI without any claim that those can delineate diagnostic entities in the medical sense, i.e. real nosological entities.

However, the data collected in our study are multi-dimensional both in space with a large number of voxels and including multiple observations per variable and highly correlated. Therefore, we have decided to complement the more conventional two sample t-tests analysis with multivariate methods, namely multivariate linear model (MLM) (13). Multivariate analysis is widely used in studies with highly-dimensional data and multiple variance. Furthermore, the method measures the strength of the relationship amongst variables and summarizes data about the individual differences. These methods have already been successfully applied to datasets from neuroimaging (14, 15) and on rather limited scale in psychiatry (16, 17).

## AIM

In this context the aim of the present study is to identify by means of multivariate analysis the underlying biological signatures comprised of brain signals which may explain the variance across clinical diagnostic measures, presented simultaneously with the acquisition of the fMRI signal, such as depression (DS) and paranoid (PS) scale scores, particularly incorporated within PD-S, and diagnostically neutral (DN) items from the same interest scale as employed in our previous studies. In this way we may foster the diagnostic validity of the clinical measures and disease entities in question.

The objective of the study is to construct a bottom-up unsupervised machine learning approach, where the brain signatures identified by three principal components based on activations yielded from the three kinds of stimuli (DS, PS, and DN) are used in order to produce cross-validation markers which may effectively predict the variance on the level of clinical populations and eventually delineate diagnostic and classification groups.

## Methods

### Subjects

We recruited 30 adult psychiatric patients with either a diagnosis of schizophrenia (n = 16, mean age 36.4 ± 12.5 y, 10 males), or depressive episode (n = 14, mean age 45.3 ± 12.5 y, five males). Subjects were assessed by an experienced psychiatrist using a comprehensive clinical interview and the structured Mini International Neuropsychiatric Interview (M.I.N.I 6.0) (18) as well as the Montgomery-Åsberg Depression Rating Scale (MADRS) (19) and the Positive and Negative Syndrome Scale (PANSS) (20). Diagnosis was based on the clinical interview, the assessment scales, and the available information from past psychiatric examinations, as well as from relatives/caregivers.

Inclusion criteria for the schizophrenic group were the following: 1) Diagnosis of Schizophrenia according to *DSM-IV TR* 2) Age 18 to 65 years. 3) PANSS total score at least 60. For the depression group subjects had to comply with the *DSM-IV TR* criteria for depressive episode (either in the context of major depressive or bipolar disorder), with MADRS score at least 20 and age between 18 and 65 years.

Patients were excluded if they had a comorbid psychiatric disorder (such as anxiety, substance related disorder), major medical illness, neurological disease, history of head trauma with loss of consciousness, or metal implants not compatible with the MRI. All participants provided a written informed consent complying with the Declaration of Helsinki and the study was approved by the university’s ethics committee.

### Data Acquisition

Patients were scanned on a 3T MRI system (GE Discovery 750w), starting with a high resolution structural scan (Sag 3D T1 FSPGR sequence), slice thickness 1 mm, matrix 256x256, relaxation time (TR) 7.2 ms, echo time (TE) 2.3, and flip angle 12°, followed by a functional scan (2D EPI sequence), with slice thickness 3 mm, matrix 64 × 64, TR 2,000 ms, TE 30 ms, and flip angle 90°.

### Paradigm

The paradigm consisted of three different active conditions and one rest condition, with a total duration of 11 min and 44 s presented in a classic block design. Each active block lasted for 32 s and contained four text statements of 8 s. The statements of the Depression Specific (DS) and the Paranoia Specific (PS) blocks were taken from the von Zerssen depression and paranoia subscales accordingly. As in our previous study (7), there were also Diagnostically Neutral (DN) blocks consisting of four statements from a questionnaire about general interests and likes. Under each written statement four possible answers (“completely true,” “mostly true,” “somewhat true,” “not true”) and the respective four response buttons (upper left, lower left, lower right, upper right) were presented. In total there were four blocks of each type, and they were alternating between the three active conditions. After each active block a 20 s resting block followed with a fixation cross in the middle of the screen (DS:_DN:_PS:_DS:_).

### Image Processing

The SPM 12 software (Statistical Parametric Mapping, http://www.fil.ion.ucl.ac.uk/spm/) was used for the processing the functional data. The images were realigned, co-registered with the structural ones, normalized to Montreal Neurological Institute space, and smoothed with a 8 mm full-width-at-half-maximum Gaussian kernel. A general linear model was defined and the F-contrast on all three conditions was derived. The F-contrast map of each participant was used in the following analysis.

### Multivariate Analysis

MLM is a method that is applied on the highly-dimensional data and creates a reduced set of features of the original data with minimal loss. The advantages of this method are threefold. First, unlike other dimension reduction methods such as principal component analysis (PCA), MLM takes into account information coming from the data (*Y*) and the information (contextual, experimental, behavioral, etc.) encoded in design matrix (*X*).

Second, MLM is specially adapted to fMRI data in particular taking into account temporal autocorrelation of the noise. Third, as MLM takes into account noise, it can be embedded into statistical framework for making inferences. We choose MLM because it is the most suited for fMRI data. The MLM analysis is implemented in the SPM toolbox Multivariate Methods for fMRI (https://github.com/LREN-CHUV/MLM).

**Figure 1:**
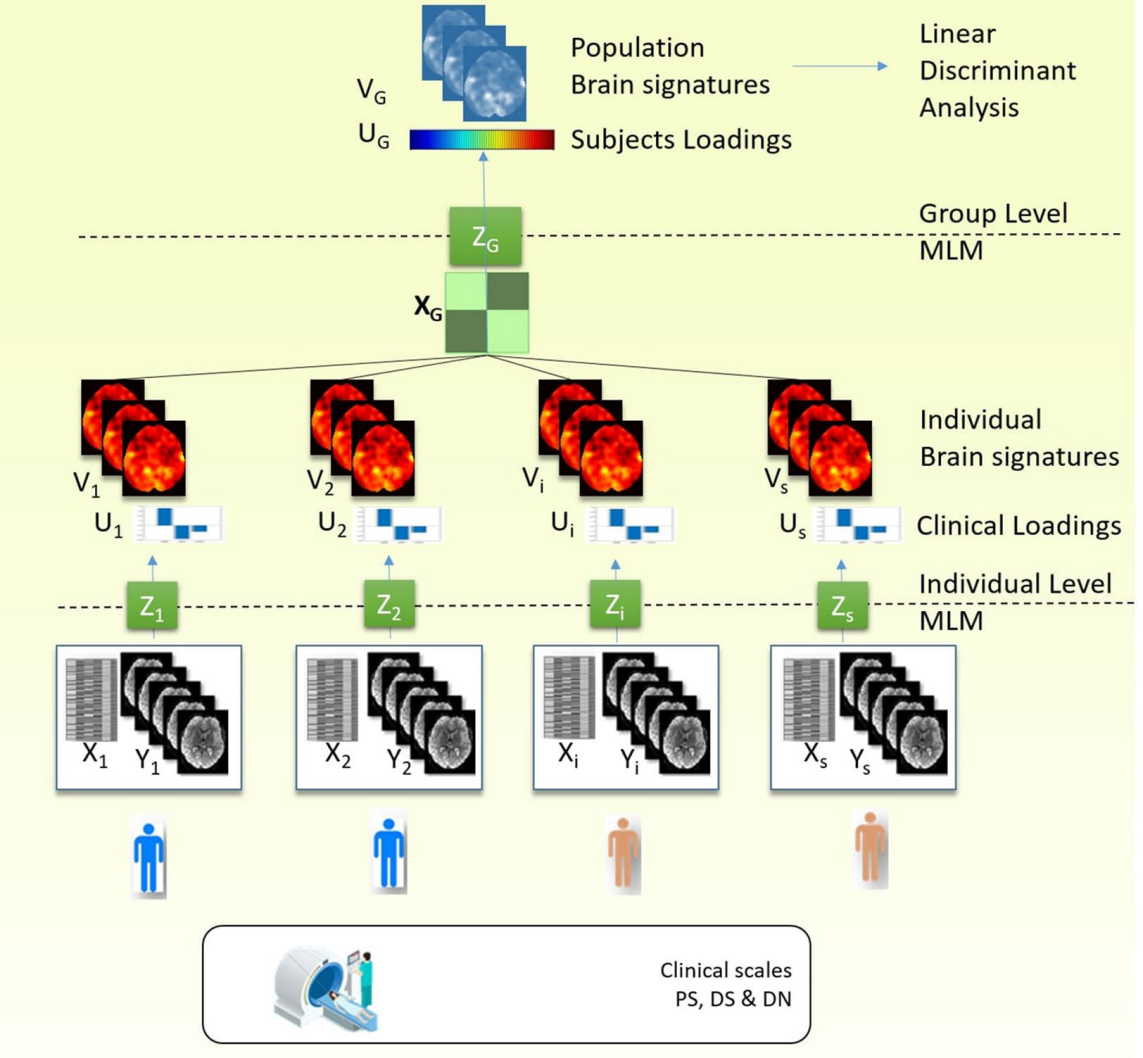
Procedure stages: 1) Individual MLM: MLM decomposed covariance matrix between the fMRI data and the design matrix which contained the clinical scale. As a result, we obtained three components (or clinical loadings) and three brain signatures (or eigenimages). 2) Group MLM: The individual eigenimages obtained from the previous step for each subject are aggregated in the group analysis, and MLM analysis is performed on the covariance matrix between eigenimages and the design matrix which contained the diagnostic label and confounding variables (gender and age). As a result we obtained group level brain signatures (or eigenimages) and the subject loadings that discriminate between the diagnostic groups. 3) To test the predictive ability of the brain signature we use linear discriminant analysis and the subject loadings to classify the individuals in two diagnostic groups and test the accuracy rates using k-fold cross-validation.

We went through the following steps in our analysis:

1. First, we performed an MLM analysis for each individual (**Figure 1**, Individual Level MLM). The individual MLM analyses identify for each participant the brain patterns that explain most of the changes in the fMRI activity and that are most correlated with the clinical conditions (PS, DS, and DN).
  Our paradigm, as described earlier, was represented in a design matrix *X* which encoded three types of stimuli (PS, DS, and DN). Nuisance covariates included the six rigid body motion parameters were also added to the design matrix.
  According to MLM algorithm, for each subject *i* (*i*=1..*s*) we calculate the principal components of matrix $Z_{i}={\left(X_{i}^{'}\Sigma_{i}X_{i}\right)}^{-1/2}X_{i}^{'}Y_{i}$, where *X* *_i_* is a design matrix [time by covariates (three conditions and nuisance covariates)], *Y* *_i_* is a data matrix (time by voxel), $X_{i}^{'}Y_{i}$ is their complex correlation normalized with ${\left(X_{i}^{'}\Sigma_{i}X_{i}\right)}^{-1/2}\;$, $\Sigma_{i}$ represents the temporal covariance matrix of the data. For each matrix *Z* *_i_* *w*e search the decomposition $Z_{i}=U_{i}\Lambda_{i\;}V_{i}^{'}$, where *U* *_i_* model parameters eigenvectors, $\Lambda_{i}$ diagonal matrix of eigenvalues, *V* *_i_* spatial eigenvectors. The model parameters eigenvectors are referred as clinical loadings and the spatial eigenvectors are referred as eigenimages. To consider only three active conditions (PS, DS, and DN), the space of interest for MLM analysis was defined by an F-contrast encompassing these condition, as mentioned earlier. As a result, we obtained three eigenimages for each subject that are used at the next step.
2. Second, to summarize the information from the individual MLMs, we then performed a second MLM analysis (**Figure 1**, Group Level MLM) using the brain patterns from the previous step while removing the confounding effects of age and gender.
  Thus, at this step we build the matrix $Z_{G}={\left(X_{G}^{'}\Sigma_{G}X_{G}\right)}^{-1/2}X_{G}^{'}Y_{G}$ where *X* *_G_* is the design matrix [subjects by covariates (diagnostic groups, age, gender)], and *Y* *_G_*=[*V*_1_,*V*_2_,…,*Vs*] is a concatenation of eigenimages (number of active conditions by voxel) of each subject. We decompose matrix $\;Z_{G}=U_{G}\Lambda_{G\;}V_{G}^{'}$. The *V* *_G_* identify the most consistent brain pattern across individuals in terms of variance explained, while to quantify individual differences we use the subject loadings *U* *_G_* (i.e. the contribution of each subject to the main brain pattern).
3. In the last step we applied a linear discriminant analysis classifier (LDA in Statistics and Machine learning toolbox, version 11.0, Matlab R2016b) on each of the three subject loadings. The purpose of this final step is to test if the brain signatures can accurately discriminate the two clinical entities. Statistical significance of the final results, meaning the ability to discriminate diagnostic groups using unthresholded brain signatures was ensured by the use of linear discriminant analysis and k-fold cross-validation. We report the accuracy of classification with receiver operating characteristic (ROC) curves.
**Figure 1** describes the schematic of our approach for discovering the brain signatures. To identify the brain signatures, we use multivariate method both at individual and group/population levels.

## Results

### Demographic and Clinical Characteristics

The two patient groups did not differ significantly in their demographic and clinical characteristics (**Table 1**).

**Table 1:** Demographic and clinical characteristics of the samples.

	Schizophrenia patients (n = 16)	Depressed patients (n = 14)	Statistical significance
Age (mean ± SD)	36.4 ± 12.5	45.3 ± 12.5	0.064^a^
Sex (M/F)	10/6	5/9	0.143^b^
Education (secondary/higher)	11/5	8/6	0.452^b^
Age at onset (years)	28.5 ± 7.7	35.9 ± 11.2	0.099^a^
Illness duration (months)	93.8 ± 84.6	145.0 ± 86.0	0.200^a^
Episode duration (weeks)	8.6 ± 6.3	11.7 ± 9.4	0.419^a^

SD, standard deviation. ^a ^Independent samples t-test, ^b^χ^2^ test.

### MLM Results

**Figure 2:**
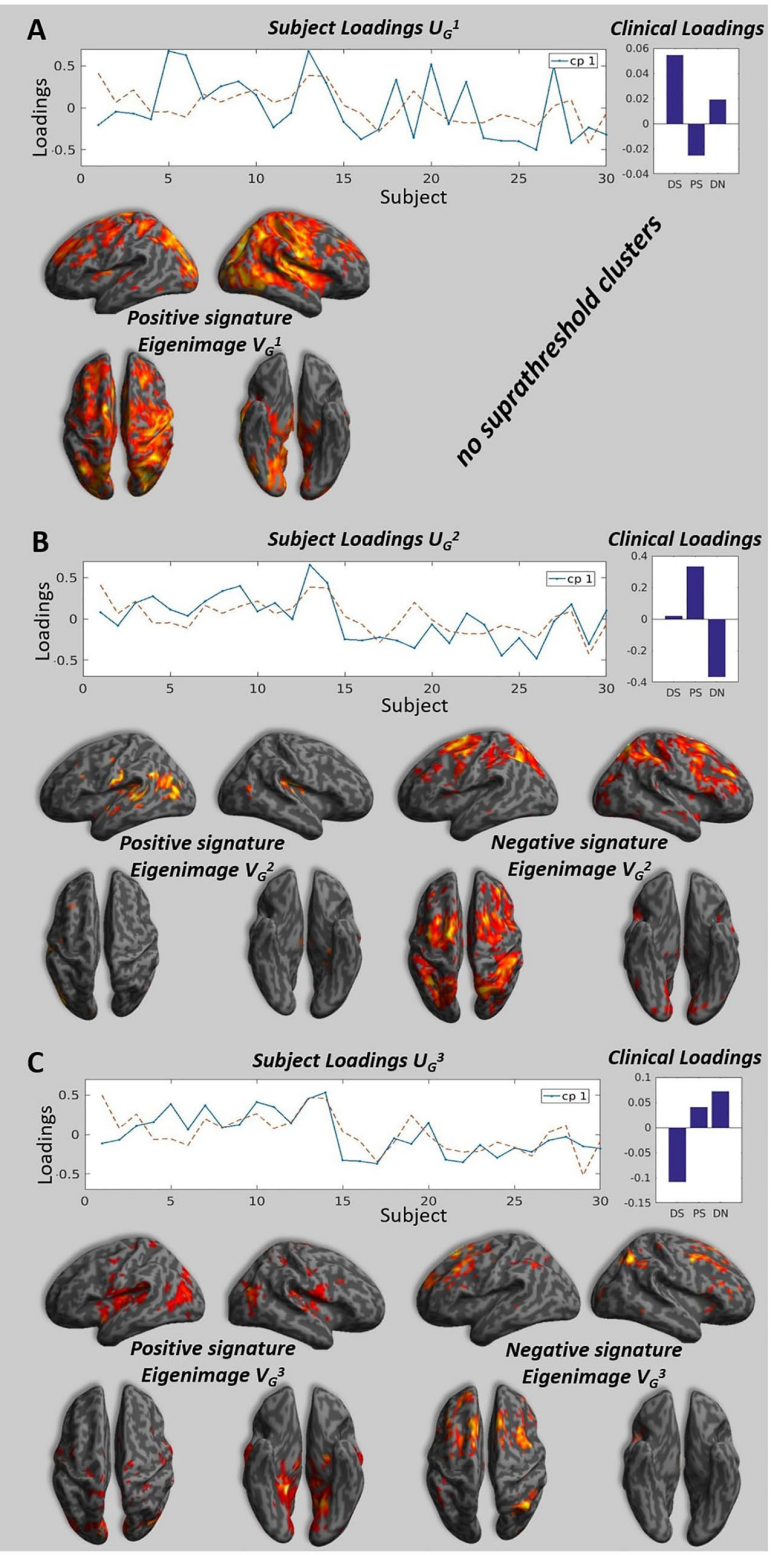
Brain signatures and subject loadings across all participants. Panels **A**, **B**, and **C** show the subject loadings for the first, second and third components, the corresponding signature and the clinical loadings. The subject loadings are shown as the solid blue line on the graph, the dotted line represents the projection of subject loadings in the design space (the units are arbitrary). The signatures represent the correlation between the subjects loadings and the value at each voxels. We project the strength of this correlation measure by a T-test on a 3D brain for illustration purposes, please note that the only valid test is the multivariate test that take into account all the voxels in the brain (see Kherif 2002 for details) and all the voxels with the appropriate weighting are taken into consideration when performing classification. The clinical loadings are the averaged clinical loadings of each subject calculated at the individual level MLM and weighted by the subject loading obtained at the group level MLM. **(A)** The highest peaks (T > 3.40, p < 0.001, uncorrected) for the positive pattern were located in the parietal cortex, precuneus, inferior occipital cortex, thalamus, interior cingulate gyrus, postcentral gyrus. There were no voxels significantly different from zero at the same threshold for negative pattern. **(B)** The highest peaks (T > 1.7, p < 0.05, uncorrected) for the positive pattern were located in the central operculum, superior temporal gyrus, and left hippocampus. The highest peaks (T > 1.7, p < 0.05) for the negative pattern were located in the superior frontal gyrus, middle frontal gyrus, angular gyrus. **(C)** The highest peaks (T > 1.7, p < 0.05, uncorrected) for the positive pattern were located in the lingual gyrus, precuneus, planum temporale, hippocampus, and insula. The highest peaks (T > 1.7, p < 0.05) for the negative pattern were located in the middle frontal gyrus, superior frontal gyrus, and angular gyrus.

The individual MLMs showed a consistent profile across the different participants (see **Figure 2**, Clinical Loadings to the right side). In all the subjects, the first component that explained most of the variance corresponds to positive loading for the DS and DN and negative loadings for PS. The second component, shows a positive loading for DS and PS and negative loadings for DN, finally the last component shows a positive loading for PS and DN and a negative loading for DS.

Following the individual and group MLM, we identified the three brain patterns that summarized all the individual variabilities of the individual brain patterns (see **Figure 2**). The first brain signature shows positive pattern that covers visual parietal, motor cortices and it also expands to the frontal lobes. The second brain signature was mostly characterized by a positive pattern in the temporal and negative pattern in the frontal and parietal lobes. Finally, the third signature had mainly medial temporal and mid-frontal contributions for the positive and negative signature respectively.

**Figure 3:**
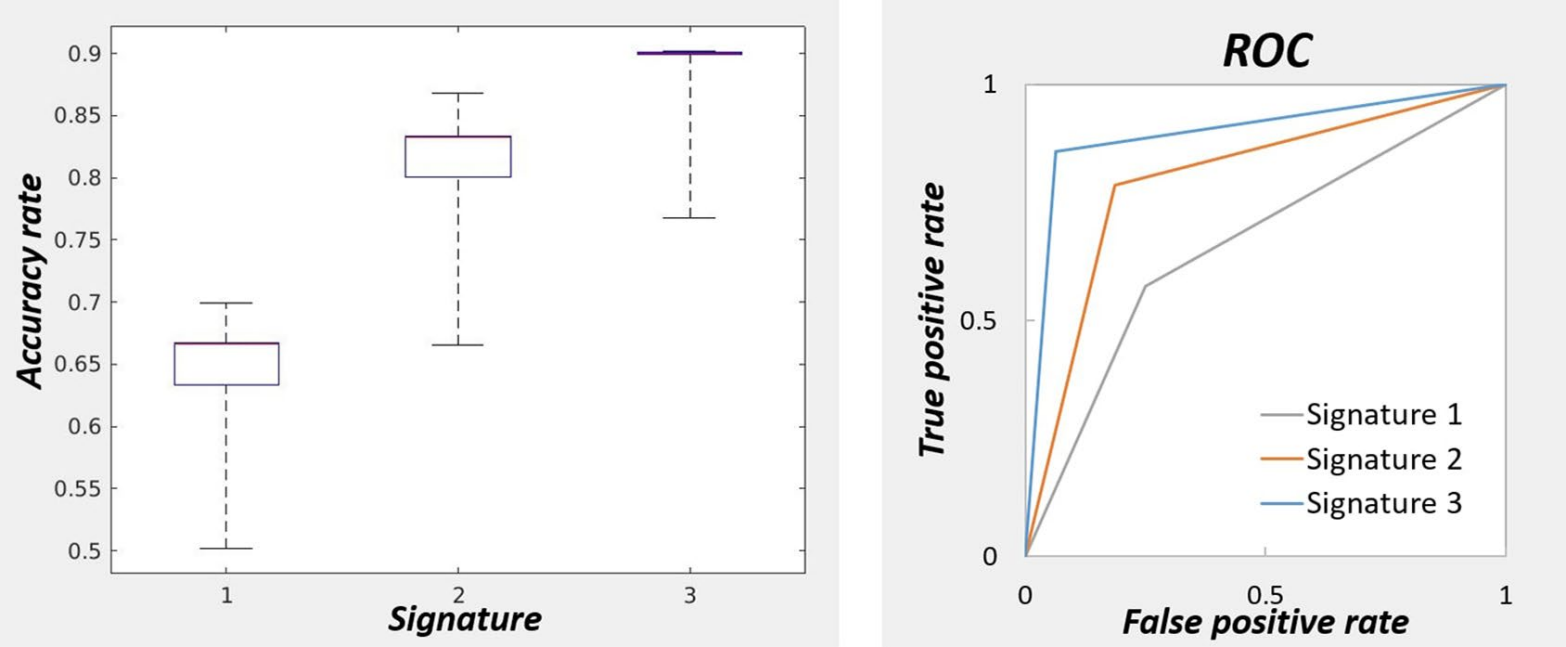
On the left: Accuracy of the classifier for three signatures for predicting the diagnostic labels. The accuracies were obtained by cross-validation repeated 100 times to obtain the percentiles. The highest accuracy was obtained with the brain signature 3. On the right: Performance measurement of the classifiers for three signatures with receiver operating characteristic curve.

**Figure 3** (left) represents the accuracy of the linear discriminant analysis on subjects’ loadings for three signatures. The signatures were taken both for positive and negative patterns without thresholding. The accuracy was measured using k-fold cross validation with *k* = 2 and repeated 100 times to estimate the medians and 25^th^ and 75^th^ percentiles of its distribution. The median accuracy was respectively 0.67, 0.83, 0.90 for the first, second, and third signatures respectively. The performance of the classifier for each signature is measured with the ROC curves using schizophrenic group as reference (**Figure 3**, right).

## Discussion

In this current research, we have been able to separate the two investigated clinical entities—schizophrenia and depression by use of brain signatures derived from a task related fMRI where the paradigm comprised of answering to a self-assessment scale. This is a confirmation of the possibility to achieve bottom-up classification of mental disorders, by use of the brain signatures relevant to clinical evaluation tests.

However, there are several methodological issues to be discussed. On one hand, the small sample size might have influenced the results. On the other hand, the paradigm used was designed to discriminate between schizophrenia and depression by means of a contrast of the BOLD signal acquired during the depression and paranoia items processing but as we know both from clinical practice and psychiatric research these two domains may overlap (21). Symptoms of depression are often seen in schizophrenia and PCA of the PANSS items has revealed an anxio-depressive component highly correlated with other depression scales Hamilton depression rating scale (HAM-D), Calgary Depression Scale for Schizophrenia (CDSS) (16). Moreover, by means of PCA of ten frequently used negative symptom scales and structural brain imaging, Chuang et al. were able to find distinct correlation between the components and the white and gray matter volumes of different regions in a group of patients with schizophrenia and depression respectively (22).

Despite these limitations our study is adding to the growing body of evidence that multivariate approaches can be reliably used for distinguishing major psychiatric disorders by their respective brain signatures. For example, patterns derived from structural MRI have been used to discriminate between schizophrenia and healthy controls with high specificity and sensitivity ranging from 80% to 90%, and a bit less than 90% for schizophrenia versus bipolar disorder, as well as around 80% when compared to major depression (23–26).

Connectivity measures have also been used to distinguish between schizophrenia and healthy controls or depressed patients with an accuracy rate of 70% to 80% (27, 28). In a multisite study on fMRI (obtained under resting or different cognitive and emotional tasks), Orban et al. were able to achieve a discrimination rate of schizophrenia patients versus healthy controls as high as 84% (29). Thus, our accuracy rate of 67% to 90% is comparable to the results stated in most of the published literature to date. However, surprisingly the first two components that explain most of the variance did not necessarily led to the highest accuracy. This highlights again the limitation of psychiatric diagnostic entities. Put simply, there is a lot of variance due to biological processes although related to the disease that do not entirely correspond to the diagnostic groups. The first two brain signatures presents high contribution of the sensory cortices (motor or visuals), the third signature shows brain patterns with high loads in the temporal, parietal, and frontal regions. Unlike previous methods our two step hierarchical approach using semi unsupervised method allows to uncover these underlying biological processes and to identify the ones predictive of the diagnostic groups.

Moreover, what distinguishes our research from similar classification studies in the field is that our paradigm is based on the application of clinically relevant evaluation tools (in this case the PD-S) not just resting state or tasks that are irrelevant to the everyday patient assessment. In this way, our approach has the potential to practically bridge the gap between neuroscience and bedside care. We believe that the current research represents an advancement of the theoretical concept of the translational validation supporting it with further empirical results (25).

In contrast to our previous study where classical contrasting of the BOLD-signal elicited by the processing of the paranoia or depression items has failed to reveal statistically significant differences between the two clinical samples (despite the apparent differences), here by means of PCA and MLM we have achieved a meaningful distinction on the group level in a bottom-up fashion. This is in support of the further use of these techniques as they might better reflect the complexity of both the neuroimaging data as such and the respective diagnostic classes.

## Conclusion

This paper is supposed to complement our previous publications (6–8) which used conventional approach for top-down cross-validation of clinical self-evaluation diagnostic scale and fMRI, with rather limited results. Here, we demonstrate that by use of the items from the same clinical scale as fMRI stimuli and the means of machine learning it is possible to discover the brain signatures behind different psychiatric diagnostic classes and respective clinical measures.

This approach may potentially encourage in future re-validation of both psychiatric classifications and methods of assessment based on more robust neuro-biological evidence.

## Data Availability Statement

The data are made available to public on the following address: https://doi.org/10.5281/zenodo.3497072. 

## Ethics Statement

The studies involving human participants were reviewed and approved by Research Ethics Committee, Plovdiv Medical University. The patients/participants provided their written informed consent to participate in this study.

## Author Contributions

DS has formulated the concept behind the study as exposed in the introduction. FK has produced the methods and results. SK delivered the discussion. RP has been reponsible for the data management and statistical analysis. AL and JB performed second level statistical analysis and generated the figures in the article.

## Funding

FK received funding from the European Union Seventh Framework Programme (FP7/2007-2013) under grant agreement number 604102 (HBP Ramp-Up Phase) and grant agreement number 720270 (HBP SGA1), the VELUX STIFTUNG and Pharnext, Paris.

## Conflict of Interest

The authors declare that the research was conducted in the absence of any commercial or financial relationships that could be construed as a potential conflict of interest.
